# Characterization of the Upper Respiratory Tract Microbiomes of Patients with Pandemic H1N1 Influenza

**DOI:** 10.1371/journal.pone.0069559

**Published:** 2013-07-02

**Authors:** Bonnie Chaban, Arianne Albert, Matthew G. Links, Jennifer Gardy, Patrick Tang, Janet E. Hill

**Affiliations:** 1 Department of Veterinary Microbiology, Western College of Veterinary Medicine, University of Saskatchewan, Saskatoon, Saskatchewan, Canada; 2 Women’s Health Research Institute, Vancouver, British Columbia, Canada; 3 Saskatoon Research Centre, Agriculture and AgriFood Canada, Saskatoon, Saskatchewan, Canada; 4 British Columbia Centre for Disease Control, Vancouver, British Columbia, Canada; 5 Department of Pathology and Laboratory Medicine, University of British Columbia, Vancouver, British Columbia, Canada; University of Georgia, United States of America

## Abstract

The upper respiratory tract microbiome has an important role in respiratory health. Influenza A is a common viral infection that challenges that health, and a well-recognized sequela is bacterial pneumonia. Given this connection, we sought to characterize the upper respiratory tract microbiota of individuals suffering from the pandemic H1N1 influenza A outbreak of 2009 and determine if microbiome profiles could be correlated with patient characteristics. We determined the microbial profiles of 65 samples from H1N1 patients by *cpn*60 universal target amplification and sequencing. Profiles were examined at the phylum and nearest neighbor “species” levels using the characteristics of patient gender, age, originating health authority, sample type and designation (STAT/non-STAT). At the phylum level, Actinobacteria-, Firmicutes- and Proteobacteria-dominated microbiomes were observed, with none of the patient characteristics showing significant profile composition differences. At the nearest neighbor “species” level, the upper respiratory tract microbiomes were composed of 13-20 “species” and showed a trend towards increasing diversity with patient age. Interestingly, at an individual level, most patients had one to three organisms dominant in their microbiota. A limited number of discrete microbiome profiles were observed, shared among influenza patients regardless of patient status variables. To assess the validity of analyses derived from sequence read abundance, several bacterial species were quantified by quantitative PCR and compared to the abundance of *cpn*60 sequence read counts obtained in the study. A strong positive correlation between read abundance and absolute bacterial quantification was observed. This study represents the first examination of the upper respiratory tract microbiome using a target other than the 16S rRNA gene and to our knowledge, the first thorough examination of this microbiome during a viral infection.

## Introduction

In 2009, a novel H1N1 influenza A strain (A(H1N1) pdm09) reached global pandemic status. It is estimated that tens of millions to 200 million people were infected with A(H1N1) pdm09 worldwide, with 2-5% of confirmed cases in Canada and the USA requiring hospitalization [1,2]. Although many hospitalizations and deaths could be attributed to recognized risk factors (cardiovascular disease, respiratory diseases, auto-immune disorders, obesity, diabetes, cancer or pregnancy), close to one third of hospitalized patients who died had no known underlying medical conditions that would have predisposed them to severe infection [1]. The last major pandemic of an H1N1 influenza A strain was the Spanish flu of 1918-19. Recent investigations into that outbreak have determined that for many patients, if not most, secondary infection with bacterial pneumonia was the major cause of morbidity and mortality [3]. Along a similar vein, current research has shown that *Streptococcus pneumoniae* coinfection is correlated with severity of A(H1N1) pdm09 influenza illness [4]. These findings highlight the importance of bacteria in the respiratory tract during a viral infection. However, research into the associations of bacteria with A(H1N1) pdm09 infections has to date focused only on known pathogens.

The normal upper respiratory tract microbiota of humans has not received the same intense study as other body sites; nevertheless, a basic picture of this community has emerged. The current consensus is that a healthy nose/nasopharynx has a bacterial community dominated by Actinobacteria and Firmicutes, with an increasing presence of Proteobacteria farther from the nose in the nasopharynx [5–8]. An inverse correlation between the prevalence of Actinobacteria and Firmicutes has been noted, with individuals having microbiomes dominated by either one phylum or the other, suggesting a possible antagonistic relationship between the groups [7]. At the genus level, similar to skin microbiomes, *Corynebacterium* spp., *Propionibacterium* spp. and *Staphylococcus* spp. are prominent members of the upper respiratory tract microbiome [6,7,9]. However, despite our growing understanding, we do not yet know if any particular upper respiratory tract microbiota compositions are associated with particular patient characteristics during a respiratory tract infection.

In the context of A(H1N1) pdm09, one study briefly examined the upper respiratory microbiota of 2009 A(H1N1) pdm09 patients while doing a metagenomic analysis of RNA from nasopharyngeal swabs of 17 individuals [10]. Although the focus of that work was to examine the viral component of the microbiome, a small fraction of bacterial ribosomal RNA (rRNA) sequences were also detected. Interestingly, the most detected bacterial families observed in individual patients were Enterobacteriaceae (Proteobacteria, 7 patients), Moraxellaceae (Proteobacteria, 4 patients), Streptococcaceae (Firmicutes, 2 patients), Carnobacteriaceae (Firmicutes, 2 patients), Pasteurellaceae (Proteobacteria, 1 patient), and Oxalobacteraceae (Proteobacteria, 1 patient) [10]. This composition is notably different from previous studies (more Proteobacteria-dominated profiles, no Actinobacteria-dominated profiles) and raises the question of whether this represents a methodological difference or an actual difference in the upper respiratory microbiota during A(H1N1) pdm09 infection compared to healthy individuals.

The goal of the current study was to characterize the organisms present in the upper respiratory tract of a range of individuals during A(H1N1) pdm09 infection. We obtained samples from A(H1N1) pdm09 patients in British Columbia, Canada during the 2009 pandemic and determined their microbiome compositions by metagenomic profiling with the *cpn*60 universal target and qPCR. We found the microbiomes clustered based on composition into a few recurring profiles that were independent of patient gender, age, originating health authority, sample type collected or its STAT/non-STAT designation. In addition, we found the number of metagenomic sequence reads obtained correlated strongly with absolute qPCR quantification for seven species that were present at a range of prevalences in the data. This confirmed that metagenomic sequence frequencies obtained from the *cpn*60 gene accurately reflected the composition of the samples.

## Materials and Methods

### Ethics statement

Samples were selected from a larger study examining the spatio-temporal distribution of A(H1N1) pdm09 across British Columbia during the 2009 pandemic. Identifying data was stripped from the sample record, leaving only patient gender, age, regional health authority, specimen type and STAT/non-STAT designation. The study was approved by the University of British Columbia Clinical Research Ethics Board (Protocols B09-0284 and H09-02695).

### Sample collection

A total of 67 clinical samples sent to the British Columbia Public Health Microbiology and Reference Laboratory (BC PHMRL) between April 24, 2009 and March 29, 2010 for influenza A testing were selected. All samples were confirmed A(H1N1) pdm09-positive by RT-PCR and sequencing at the BC PHMRL. Samples were collected by physicians at clinics and hospitals in the Fraser (n=11), Northern (n=13), Vancouver Island (n=8), Vancouver Coastal (n=12) and Interior (n=23) health authorities in British Columbia, Canada. Clinical specimens were chosen for the microbiome study to reflect the type and proportion of samples received for H1N1 testing by the BC PHMRL and included nasal swabs (n=23), nasopharyngeal swabs (n=32), nasopharyngeal washes (n=11) and tracheal aspirates (n=1). Patients included both genders (male=33, female=33, undefined=1) and ranged in age from <1 to 89 years. Samples were collected in hospitals (“STAT” designation, n=35) or non-hospital, community settings (“non-STAT”, n=32) (Table S1).

### Nucleic acid extraction

Samples were received at the BC PHMRL through standard clinical sample submission protocols. Total nucleic acid was extracted from clinical material by MagMAX™ Viral RNA Isolation Kit (Life Technologies, Burlington, ON, Canada). Isolated nucleic acid (DNA and RNA) was precipitated for storage and transport. Nucleic acid was re-suspended in 100 µl of TE buffer (10 mM Tris-HCl, 1 mM EDTA; pH 8.0) prior to analysis.

### *cpn60* universal target (UT) PCR and pyrosequencing

The universal target (UT) region of the *cpn*60 gene was amplified using a primer cocktail consisting of a 1: 3 molar ratio of primers H279/H280:H1612/H1613 (Table 1) [11–13]. Primer sets were modified at the 5' end with 10-mer multiplexing identification (MID) sequence as per pyrosequencing recommendations to facilitate multiplexing of samples for sequencing (Roche, Brandford CT, USA). PCR reactions consisted of 1 × PCR reaction buffer (20 mM Tris-HCl (pH 8.4), 50 mM KCl), 2.5 mM MgCl_2_, 200 µM dNTP, 800 nM of primer cocktail, 2.5 U Platinum Taq DNA Polymerase (Invitrogen, Burlington, ON, Canada) and 2 µl template DNA, in a final volume of 50 µl. Twelve PCR reactions were run for each sample in a thermocycler (Eppendorf Mastercycler) over a temperature gradient: 94°C for 3 min, followed by 40 cycles of 30 sec at 94°C, 1 min at 42-60°C and 1 min at 72°C, followed by a final extension at 72°C for 10 min. PCR reactions from the same sample were pooled and concentrated using the AMPure Purification system (Agencourt Bioscience, Beverly, MA, USA), and further purified by agarose gel separation and extraction (QIAEX II gel extraction kit, Qiagen, Toronto, ON, Canada). Final amplicon was suspended in TE buffer and quantified using a Qubit fluorometer (Invitrogen). *cpn*60 UT amplicon libraries were pooled in equimolar concentrations and sequenced using the GS FLX Titanium system as per manufacturer’s instructions.

**Table 1:** PCR primers and conditions used in this study.

**Target**	**Primer name**	**Primer sequence** ^a^	**Anneal (**°C**)**	**Reference**
*cpn*60	H279	GAIIIIGCIGGIGAYGGIACIACIAC	40-60	[11]
	H280	YKIYKITCICCRAAICCIGGIGCYTT		
	H1612	GAIIIIGCIGGYGACGGYACSACSAC		
	H1613	CGRCGRTCRCCGAAGCCSGGIGCCTT		
*Corynebacterium accolens* (Ca)	JH0366	AGAGTCCAATACCTTCGG	62	This study
	JH0367	CTCCTGACGCTCCATATC		
*Corynebacterium* *pseudodiphtheriticum* (Cp)	JH0374	TGAACAAGGATTCCGTTA	58	This study
	JH0375	CAGGATGTATGGGTCTTC		
*Enterococcus* *hirae* (Eh)	JH0144	TGCTCAAGTTGCAGCTGTTT	62	This study
	JH0145	TTCGGTTTCGATTCCTTTTG		
*Staphylococcus aureus* (Sa)	JH0399	CTGAACTAGAAGTGGTTGAAGGT	67	This study
	JH0400	CGCATACGGTTTAGCACGATA		
Streptococcus pneumoniae (Sp)	JH0380	ACGCAATCTAGCAGATGAAGCA	60	[22]
	JH0381	TCGTGCGTTTTAATTCCAGCT		
	JH0382(probe)	(*FAM*)TGCCGAAAACGCTTGATACAGGGAG(*BHQ1*)		
Streptococcus mitis (Sm)	JH0376	GCCGTCTCTTCTCGTTCT	62	This study
	JH0377	GGATTTTCAAGATCAGCTACCATT		
*Propionibacterium acnes* (Pa)	JH0372	TATATCCTTATCGTCAACTCCAA	62	This study
	JH0373	CTCGGCAATGACAAACAG		
16S rRNA gene (16S)	SRV3-1	CGGYCCAGACTCCTAC	62	[23]
	SRV3-2	TTACCGCGGCTGCTGGCAC		
Human cytochrome C oxidase subunit 1 (*cox1*)	JH0241	CACCTTCTTCGACCCCGCCG	67	This study
	JH0242	TGCTTCCGTGGAGTGTGGCG		

^a^ I = inosine, Y=C or T, R=G or A, K=G or T, S=G or C, FAM = Carboxyfluorescein, BHQ1 = Black hole quencher 1

### DNA sequence quality control and assembly of OTU

Pyrosequencing data was processed using the default on-rig procedures from 454/Roche. Filter passed reads were assessed for non-*cpn*60 host DNA by screening against a database comprised of the human genome with annotated *cpn*60 genes and predicted pseudo-genes removed. MID-partitioned sequences were processed with the microbial Profiling Using Metagenomic Assembly (mPUMA) pipeline (http://mpuma.sourceforge.net [14]) to generate operational taxonomic units (OTU) with gsAssembler (Roche). OTU were screened and filtered for chimeras with Chaban’s Chimera Checker (C3) (http://mpuma.sourceforge.net) and Bellerophon [15]. Remaining OTU were identified by watered-Blast comparison [12] to the *cpn*60 reference database, cpnDB_nr (downloaded from www.cpndb.ca [16]). OTU abundance was calculated based on mapping of sequence reads to OTU sequences using Bow tie 2 in mPUMA. OTU having the same database reference were collapsed into nearest neighbor “species” and OTU with less than 55% identity to any reference sequence were removed from the dataset as non-*cpn*60 sequence.

### Community composition analysis

A number of parameters and characteristics were calculated for each sample’s microbial community profile. Good’s coverage estimates were determined using mothur [17]. Shannon’s diversity index, Simpson’s diversity index, Chao1 estimate, the number of observed species and Jackknifed beta diversity were calculated with Quantitative Insights Into Microbial Ecology (QIIME) software [18]. Phylogenetic-based distance matrices and clusters were examined using Unifrac [19] and a Bray-Curtis dissimilarity matrix was computed in R using the vegan package [20,21].

### Quantitative PCR (qPCR)

Absolute quantification of bacterial species of interest in each sample was done by qPCR using primers and annealing temperatures described in Table 1. Quantification of *Streptococcus pneumoniae* was achieved by detection of the *lytA* gene using a Taqman assay [22], while all other species-specific assays were SYBR Green-based, targeting the *cpn*60 gene. SYBR Green assays were also used for total 16S rRNA gene content estimation based on amplification of the V1-V3 region of the 16S rRNA gene [23], while an estimate of human mitochondrial DNA content was based on quantification of the cytochrome C oxidase subunit 1 (*cox1*) gene.

For all assays designed in this study (Table 1), the *cpn*60 sequence for each target species type strain was used to design species-specific primers. Signature regions unique to each target were determined using Signature Oligo software (LifeIntel Inc., Port Moody, BC, Canada), primers were designed with Beacon Designer software (Premier Biosoft International, Palo Alto, CA, USA) and PrimerBLAST [24]. Primers were used to amplify target sequence from patient DNA samples, which were then ligated into pGEM-T-Easy (Invitrogen) to make standards and facilitate confirmation of the target by sequencing. Specificity of all primer pairs was tested by using standard plasmids from other assays as template and no products were generated. Finally, the optimal annealing temperature for each assay was determined via an annealing temperature gradient run and analysis.

All qPCR reactions were run on a plate containing a no template control (NTC) and a standard curve composed of target-containing plasmids at concentrations of 10^0^ to 10^7^ copies/reaction. All reactions were performed in duplicate. Each reaction consisted of 1 × iQ SYBR Green Supermix (BioRad, Mississauga, ON, Canada), 400 nM each primer and 2 µl of template DNA, in a final volume of 25 µl. A MyiQ thermocycler (BioRad) was used for all reactions with the following program: 95°C for 3 min, followed by 40 cycles of 95°C for 15 sec., annealing temperature (Table 1) for 15 sec., 72°C for 15 sec., and a final extension at 72°C for 5 min. A dissociation curve was subsequently run for 81 cycles at 0.5°C increments from 55°C to 95°C for 30 sec at each time point. Fluorescent signals were measured every cycle at the end of the annealing step and continuously during the dissociation curve data collection. The Taqman assay was run under identical conditions, with the substitution of 1× iQ Supermix (BioRad) in place of the SYBR Green Supermix, the addition of 200 nM JH0382 probe and the omission of the dissociation curve. All resulting data was analyzed using iQ5 Optical System Software (BioRad).

Correlations between quantified PCR copies and sequencing read abundance were calculated with SPSS software (SPSS Inc., Chicago, IL, USA).

## Results

### Pyrosequencing and quality control

*cpn*60 UT amplicon was generated from all 67 samples. Amplicons were pooled into four libraries and each was sequenced on a half plate region of a Roche 454 GS FLX Titanium pyrosequencer to generate a total of 1,940,006 filter passed sequence reads. Since previous studies of upper respiratory samples report human genomic DNA as an overwhelming proportion of nucleic acid isolated from this environment [10,25], all filter passed sequences were screened against a version of the human genome that had all *cpn*60 genes and pseudo-genes removed. This screening process filtered out 1,661,090 sequences (85.6% of the data). The remaining sequences were MID-separated and loaded into gsAssembler for *de novo* assembly. OTU sequences were then filtered to remove chimeras and non-*cpn*60 sequences (those with <55% identity to any reference database sequence). OTU abundance was calculated by mapping all reads on to the OTU using Bow tie 2 within the mPUMA pipeline. The final dataset contained 270,284 sequences in 490 OTU (Table S2). When each OTU was compared to the reference database (cpnDB_nr), the median percent identify of OTU to reference sequences was 96.4%, with 369/490 (75%) of the OTU having identities of >90% to a reference sequence (Table S3). To further aggregate the data, OTU with the same nearest neighbor (termed nearest neighbor "species") were combined. An average of 4,158 sequence reads per sample (median 1054) was obtained. Samples flu36 and flu48 contained fewer than 100 reads after processing and were removed from the analysis. Raw sequence data files from the 65 samples used in the study have been deposited to the NCBI Short Read Archive, and are associated with the BioProject accession PRJNA200951.

### Sequencing depth cut-off

An initial cut-off of at least 100 high-quality *cpn*60 sequence reads per sample was imposed on the dataset before analysis, which resulted in two samples being removed from the study. To evaluate whether this cut-off was sufficient to allow robust analyses, jackknifed beta diversity was calculated by generating 1000 rarefractions of the data at a depth of 100 reads for each of the remaining 65 samples. A distance matrix was created for each rarefaction and principal coordinates analysis (PCoA) was computed on each rarefied matrix using two different distance measures: Bray-Curtis and Canberra. Each sample was then plotted as a point where the area of each point reflected the interquartile range of the jackknifed PCoA estimates (Figure S1). The relatively compact size of the points for each sample indicated that the distance matrices were fairly consistent over 1000 iterations at the sampling depth of 100 reads, suggesting the sampling depth was adequate for sample comparison. As such, the 65 samples profiled that exceeded this minimum were used for all further analyses.

### Diversity and overall composition measures

Since it is well-established that diversity statistics are influenced by different sampling depths [26], values for the Shannon’s diversity index, Simpson’s diversity index, Chao1 estimate and the observed number of species per sample were all derived from the mean value of 1000 iterations of a 100 read subsampling of nearest neighbor “species” values in each sample. The diversity indices were then compared by patient gender, age, health authority, sample type and STAT/non-STAT designation by ANOVA (Table S4). Microbiomes contained an average of 9-14 nearest neighbor “species” when examined at the 100 read subsampling depth, and were predicted by the Chao1 estimate to contain 13-20 “species” if sampled to completion. There was no significant difference in the number of species observed or predicted between any patient characteristic groups. Diversity measurements of richness and evenness determined by Shannon’s and Simpson’s indices also showed no statistically significant difference by patient characteristics. However, there was a trend for both measures to increase as patient age increased (Shannon’s, *p* = 0.08; Simpson’s, *p* = 0.11; Table S4), with an observable distinction between patients under and over 20 years of age.

Unifrac clustering, considering both phylogenetic composition of the microbiome and normalized sequence read abundance of the 215 nearest neighbor “species” sequences in each sample, was performed (Figure S2). There were no meaningful groupings of upper respiratory tract microbiomes based on our five patient characteristics. As such, there appeared to be no major phylogenetic differences in the microbiome compositions between patients based on these variables.

### Phylum-level comparisons

**Figure 1:**
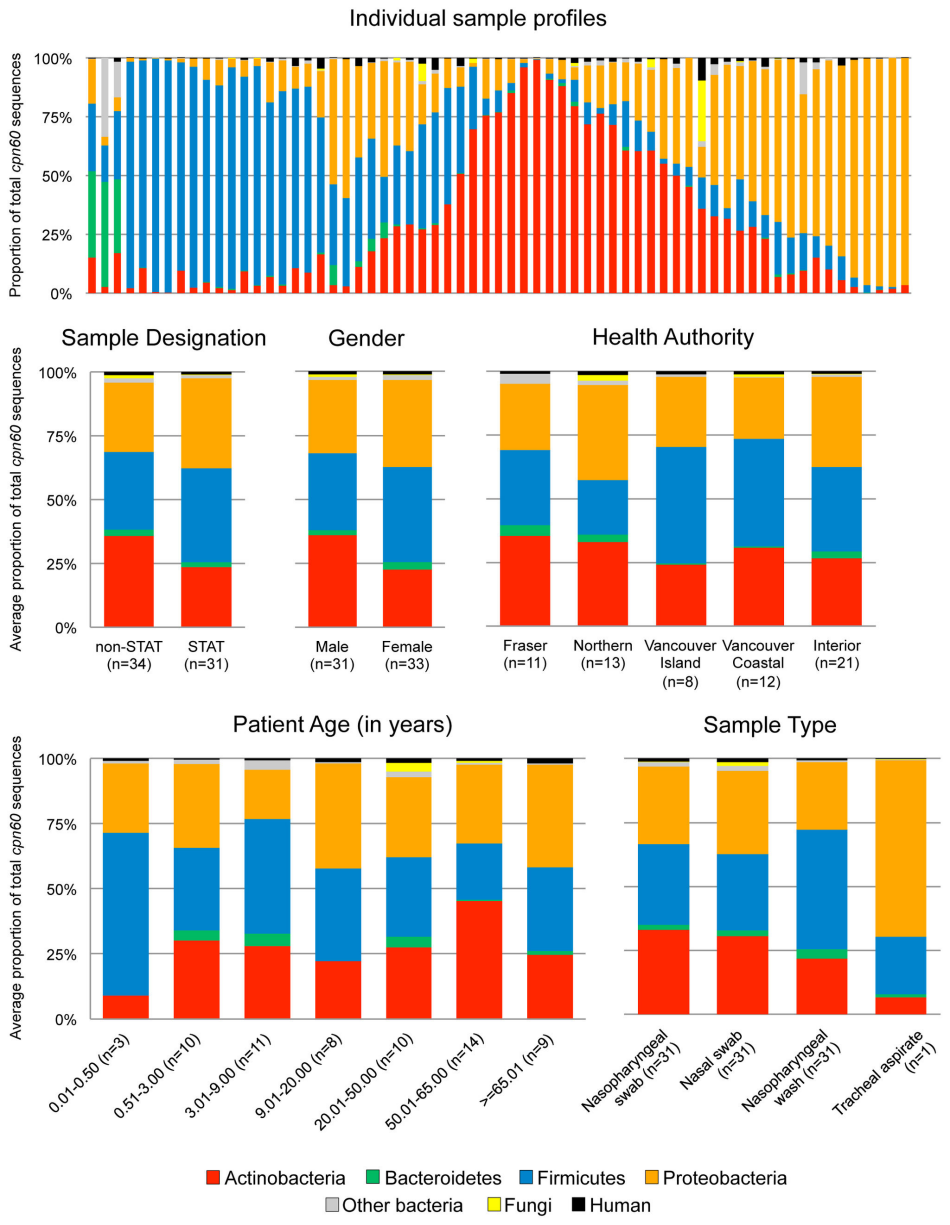
Sample composition at the phylum level. Phylum level profiles are shown for individual patients (n=65) as well as the average phylum proportions for sample designation (STAT/non-STAT), gender, health authority, patient age (in years) and sample type.

The phylogenetic composition of the combined upper respiratory tract microbiome dataset consisted of Firmicutes (42.5%), Proteobacteria (27.7%), Actinobacteria (21.7%), *Bacteroides* (5.5%), Fungi (0.1%), Human (0.2%) and other bacteria (2.3%) (Figure S3). Individual microbiomes were dominated by either Firmicutes, Actinobacteria or Proteobacteria (Figure 1), consistent with previous reports of human upper respiratory tract microbiomes [5–8].

To identify if a particular microbiome composition was associated with a patient characteristic, the proportion of each phylum in each sample was calculated and samples from the same group were averaged together (Figure 1). No statistically significant differences were observed in the microbiome compositions grouped by any variable investigated.

### Species-level microbial comparisons

**Figure 2:**
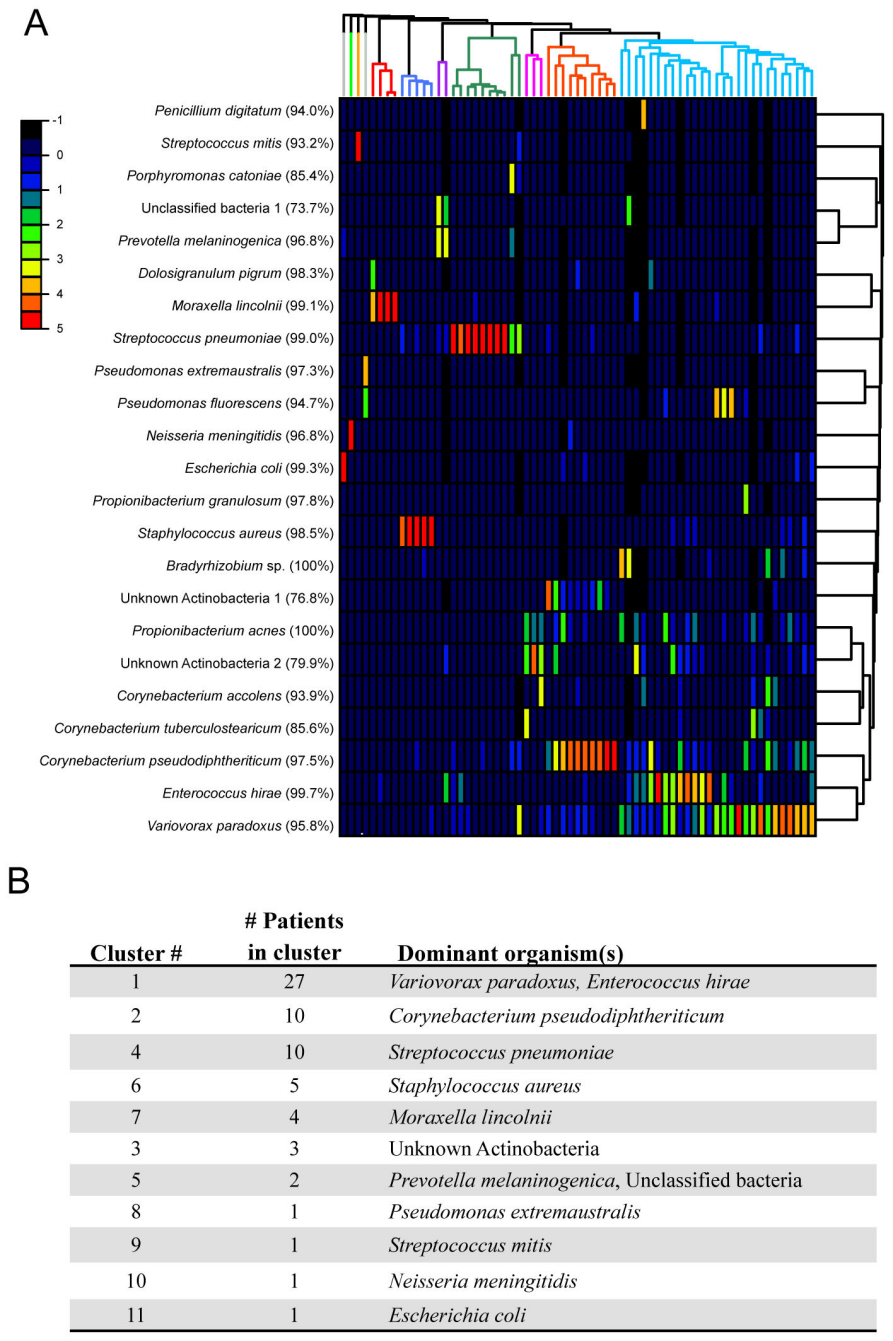
Hierarchical clustering of sample profiles. (A) Nearest neighbor “species” composing 20% or more of the microbiome in at least one sample are shown. The average percent identities of sequence reads within each nearest neighbor are given in parenthesis. The color scale (black to red) reflects an increasing relative abundance of sequence reads for each nearest neighbor “species” in each sample. (B) Summary of the hierarchical clustering detailing the number of samples and dominant organism(s) in each cluster.

To investigate the microbiome at a finer taxonomic resolution, the nearest neighbor “species” description of each sample was used to construct a Bray-Curtis dissimilarity matrix. The matrix was calculated by converting the number of sequence reads for each “species” in a sample to its relative abundance within that sample. The jackknife beta diversity analysis results (Figure S1) suggested that the distances between samples were fairly robust, so the actual sequence counts, rather than rarefied numbers, were used for this analysis. Average linkage hierarchical clustering of the dissimilarity matrix generated 11 clusters (Figure 2). There were five clusters consisting of only one or two patient samples (clusters 5, 8, 9, 10 and 11). Cluster 1 was the largest, with 27 patients with *Variovorax* *paradoxus* and *Enterococcus* *hirae* as the dominant organisms. Clusters 2 and 4 were the next largest, each containing 10 patients, with *Corynebacterium* *pseudodiphtheriticum* and *Streptococcus pneumoniae* as the dominant organisms, respectively.

### Absolute quantification of targets

To assess the reliability of sequence read abundance from pyrosequencing of the *cpn*60 gene, we chose seven species from the dataset to evaluate by quantitative real-time PCR (qPCR). This set was comprised of the three most abundant species in the dataset (*Staphylococcus aureus, Streptococcus pneumoniae* and *Corynebacterium* *pseudodiphtheriticum* ), two species representing 1-2% of the dataset ( *Enterococcus* *hirae* and *Propionibacterium acnes*) and two species present at less than 1% of the dataset (*Streptococcus mitis* and *Corynebacterium accolens*) (Table 2). All seven of these species were also detected in at least 31/65 samples. In addition to the individual species, estimates of total bacterial and human DNA were also obtained for comparison. qPCR was used to quantify the amount of each target of interest in the clinical sample nucleic acid extracts. A well-established Taqman assay targeting the *lytA* gene was used for quantification of *Streptococcus pneumoniae* [22]. For the remaining species, specific assays were developed for this study using SYBR Green chemistry and targeting the species type strain *cpn*60 sequence (whenever available). Assays were also designed to estimate the amount of total bacterial DNA (via quantification of the 16S rRNA gene content with primers designed previously [23]) and human DNA present (via quantification of human mitochondrial cytochrome C oxidase subunit 1 gene (*cox1*)).

**Table 2:** Correlation between normalized pyrosequencing read abundance and qPCR absolute quantity by Spearman’s rho coefficient.

**Target species**	**Proportion of total dataset (%)**	**Sample prevalence** ^a^	**Correlation between read abundance & qPCR**
*Staphylococcus aureus*	19.08	61	ρ = 0.325, p = 0.008
*Streptococcus pneumoniae*	14.60	51	ρ = 0.469, p = 0.000
*Corynebacterium* *pseudodiphtheriticum*	12.98	43	ρ = 0.475, p = 0.000
*Enterococcus* *hirae*	2.18	52	ρ = 0.432, p = 0.000
*Propionibacterium acnes*	1.13	53	ρ = 0.354, p = 0.004
*Streptococcus mitis*	0.69	31	ρ = 0.239, p = 0.055
*Corynebacterium accolens*	0.39	36	ρ = 0.458, p = 0.000

^a^ Number of samples (out of 65 total), based on sequence read abundance.

**Figure 3:**
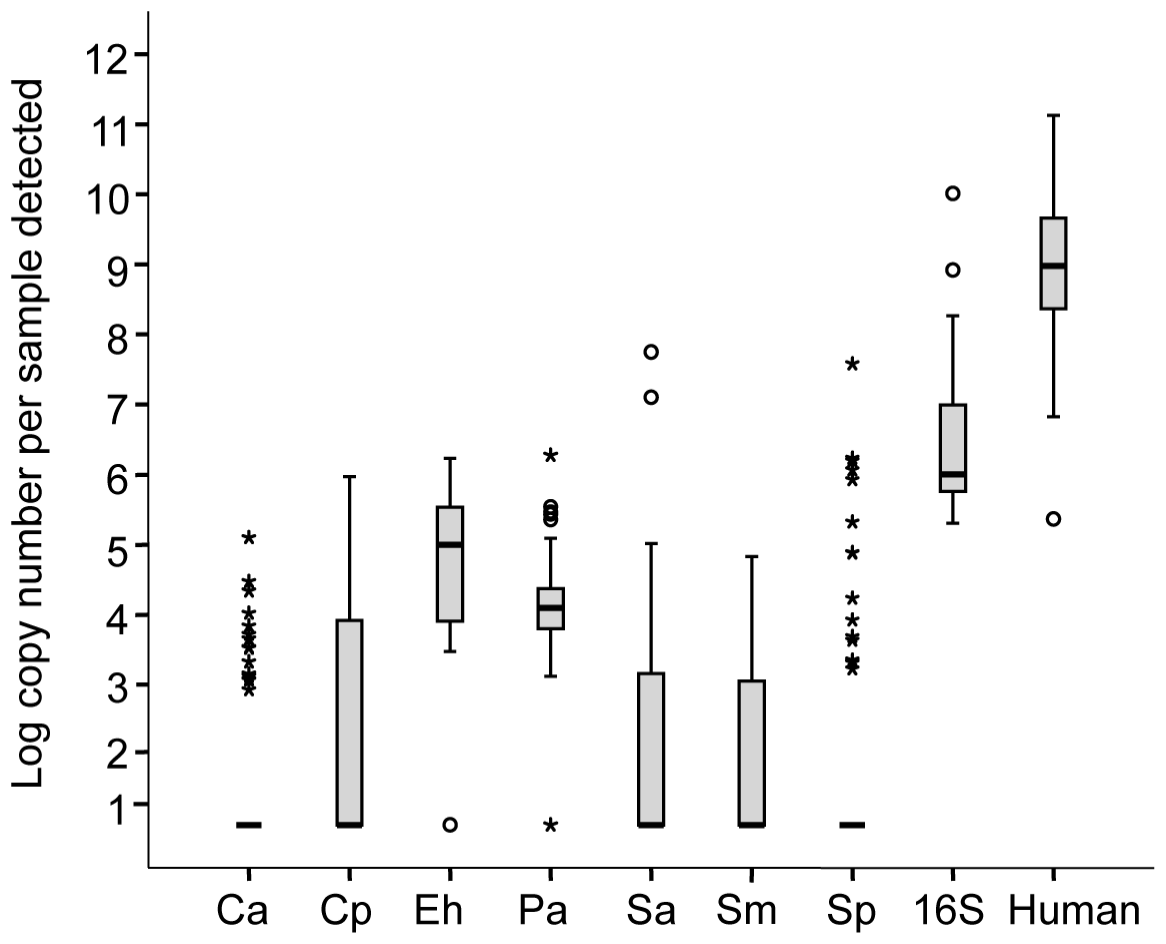
Quantification of bacterial and **human mitochondrial DNA.** Box and whisker plots showing target copies per sample detected (n = 65). Ca - *Corynebacterium accolens*; Cp - *Corynebacterium* *pseudodiphtheriticum* ; Eh - *Enterococcus* *hirae* ; Pa - *Propionibacterium acnes*; Sa - *Staphylococcus aureus*; Sp - *Streptococcus pneumoniae*; Sm - *Streptococcus mitis*; 16S -16S rRNA gene; Human - human cytochrome C oxidase gene.

The numbers of copies per sample of all targets were determined (Figure 3). The human *cox1* gene had, on average, an abundance 2.6 logs higher that the bacterial 16S rRNA gene (Figure 3, Human compared to 16S). The expected copy number of cox 1 per cell is 50-2000 based on estimates of approximately 42 mitochondria per mammalian epithelial cell [27] and 1-50 mitochondrial genomes per mitochondrion [28]. An average of approximately 1.0×10^8^ copies of *cox1* were detected per sample, which translates into 5.0×10^4^ to 2.0×10^6^ epithelial cells per sample (Figure 3). The average 16S rRNA copy number per sample was 2.5×10^5^ (Figure 3), which translates to approximately 7.5×10^5^ bacteria per sample based on a median 16S rRNA copy number of three per bacterial genome [14]. Finally, taking into consideration that an epithelial cell has significantly more DNA than a bacterial cell, it is evident that the standard clinical upper respiratory tract samples used in this study were overwhelmingly dominated by host DNA.

### Comparing sequence read abundance to absolute quantification

To determine how well pyrosequencing read abundance reflected absolute quantities per sample for the species targeted by qPCR, sequence abundance counts were compared to qPCR target copies per sample using a Spearman’s rho correlation (Table 2). With the exception of *Streptococcus mitis*, all species had a significant positive correlation (*p* < 0.05) between their sequence read abundance and their absolute quantification values. The *S. mitis* correlation was just outside the range of significance (*p* = 0.055) and could be due to qPCR assay biases, such as being too specific (missing natural species variation) or not specific enough (cross-reacting with other organisms). Alternatively, the original *cpn*60 UT PCR may not have been optimal for this species and *S. mitis* amplicons may have been underrepresented in the sequencing data.

## Discussion

Our objective was to characterize the upper respiratory tract microbiomes in patients with A(H1N1) pdm09 influenza A and determine if patient age, gender, health authority, sample type and whether the sample was collected in a hospital or not were associated with differences in microbiome composition. To determine this, the first step was to determine the microbiomes of patients confirmed A(H1N1) pdm09 positive. To facilitate the exploitation of any informative differences in future diagnostics, we chose samples for this study from specimens submitted to the BC PHMRL for routine influenza diagnostic testing. This meant that the samples were from a range of health authorities across the province, collected from a spectrum of patients in terms of gender, age, preexisting conditions, and that sample type varied from nasal swabs to nasopharyngeal swabs to nasopharyngeal washes and one tracheal aspirate. An important part of our study was to determine whether this heterogeneous sample pool (which would be typically received by a diagnostic laboratory) would yield useful information about the upper respiratory tract microbiome.

Bacterial pneumonia is a major cause of morbidity and mortality associated with influenza [3], and *Streptococcus pneumoniae* coinfection has been previously implicated in A(H1N1) pdm09 influenza illness severity [4]. Our ability to assess patient illness severity in this study was limited. During the pandemic, the BC PHMRL established a system to prioritize testing for patients with more severe illness where samples submitted for influenza testing from patients hospitalized for respiratory illness, severe respiratory infection or having lower respiratory tract symptoms were to be marked with a “STAT” designation. The “non-STAT” samples in our study were collected in a community setting from patients well enough to see a physician at a private office or clinic, and submitted for influenza testing as routine samples. However, as the pandemic progressed, almost all samples from patients attended to at the hospitals were designated “STAT”, regardless of disease severity. As such, we were unable to separate mildly and severely ill patients confidently, and could only use this characteristic as an indicator for the clinical setting in which the sample was collected.

No statistically significant differences were detected between phylum level upper respiratory tract microbiomes generated in this study when data was grouped by any of the five patient and sample variables examined (Figure 1). There were no significant differences by ANOVA comparing upper respiratory tract microbiome diversity statistics such as observed species, Chao1 estimates or Shannon’s and Simpson’s diversity index when compared using these variables (Table S4). As well, at a phylogenetic level, Unifrac failed to cluster microbiome compositions by these metadata parameters (Figure S2). The only trend noted was a moderate difference in microbiome richness and diversity between younger (<20 years) and older (>20 years) patients (Table S4). In fact, the finding that both men and women from birth to over 65 years of age share a remarkably similar collection of upper respiratory tract bacteria and fungi is impressive and notably different from what has been observed for the microbiota of other body sites [29].

The upper respiratory tract microbiome profiles generated in this study are somewhat consistent with previous culture-independent surveys generated from the nose/nasopharynx of healthy individuals (Figure 1). Frank et al. used the V1-V4 region of the 16S rRNA gene to profile the bacterial communities in the left and right nares of five healthy and 44 hospitalized individuals. Generating an average of 268 sequence reads per sample, they determined the major phyla present in the noses of healthy individuals to be Actinobacteria (68%), Firmicutes (27%), Proteobacteria (4%) and Bacteriodetes (1.4%), while hospitalized individuals had Firmicutes dominant (71%) profiles, with proportionately fewer Actinobacteria (20%) [6]. Lemon et al. profiled the nasal communities from seven healthy individuals by amplifying the full-length 16S rRNA gene from samples and identified community members by PhyloChip analysis. They observed an inverse correlation between the proportions of Firmicutes and Actinobacteria detected in an individual’s nostril microbiome. When Staphylococcaceae (Firmicutes) proportions were high, Corynebacteraceae and/or Propionibacteriaceae (Actinobacteria) proportions were low and vice versa [7]. Our dataset of 65 patients, with an average of 4158 sequences per sample, contained many samples dominated by Actinobacteria or Firmicutes, but also contained several profiles dominated by Proteobacteria (Figure 1). Although Proteobacteria were clearly seen in the previous studies, our Proteobacteria proportions were notably higher. At lower taxonomic levels (family, genus, species), many major organisms detected from our A(H1N1) pdm09 study subjects ( *Corynebacterium* *,* *Propionibacterium* *,* *Staphylococcus* and *Streptococcus* ) are comparable to previous surveys [5–8]. In addition, our dataset also contained several Proteobacteria genera, such as *Variovorax* and *Moraxella* , not as commonly reported in this environment by 16S rRNA gene-based studies.

The only study to date that has investigated the upper respiratory tract microbiome from A(H1N1) pdm09 influenza A patients was Greninger et al., during their RNA metagenome profiling study [10]. Bacterial data in this study was limited to rRNA gene sequence obtained from Illumina sequencing of total cDNA libraries. Interestingly, their finding of Proteobacteria-dominated microbiomes was mirrored in our results. The two bacterial families that dominated the microbiomes of over half of their patients, Enterobactericeae (7/17 patients) and Moraxellaceae (4/17 patients), were seen as the dominant families for Clusters 11 and 7 in our data, respectively (Figure 2). Collectively, these finding lend weight to a hypothesis that influenza A infection may be associated with a more Proteobacteria-dominated upper respiratory tract microbiome. However, additional studies addressing this hypothesis are needed before any definitive statements can be made.

One new avenue our study explored was the composition of eukaryotic members of the upper respiratory tract microbiome. While all previous studies used the 16S rRNA gene as a target, *cpn*60 has the advantage of being present in both bacteria and eukaryotes, making simultaneous detection possible. In these samples, Fungi did not represent a large proportion of the sequence reads obtained (0.1%), however three fungal species were identified, including *Malassezia* *globosa* (skin organism known to cause dandruff [30]), *Penicillium* *digitatum* (the cause of green mold on citrus fruit [31]) and *Phanerochaete chrysosporium* (environmental organism that decomposes wood [32]) (Table S3). Whether these fungi were just transients in the upper respiratory tract or whether they play a role in the microbiome is currently unknown.

Another technical aspect we sought to investigate was the reliability of sequence read counts as a marker of actual organism abundance. Evidence has been accumulating in the literature highlighting PCR, sequencing and data processing limitations that generate misleading and artifactual estimates of taxon abundance based solely on sequencing read abundance counts from metagenomic profiles [26,33,34]. The *cpn*60 UT region has been shown to alleviate some of these biases with strategic primer design [11,35], however, this is the first study to employ *cpn*60 for upper respiratory tract analysis, and so a technical examination of its performance was desired. We were surprised that 85% of our sequence data ended up matching the human genome, suggesting a significant amount of non-target amplification. The *cpn*60 PCR protocol used in this study has been previously applied to clinical samples from feces and vaginal swabs with no problems in specificity [36,37]. This led us to evaluate the relative proportions of human to bacterial DNA in these upper respiratory tract samples (Figure 3). Given that the human DNA component of these samples was an order of magnitude higher than the bacterial component, we suspect that the lower range of the temperature gradient component of the *cpn*60 universal PCR (originally designed for maximum amplification of diverse bacteria [11]) allowed for the non-specific amplification. Studies are underway to evaluate this parameter and address this issue for future studies involving samples dominated by host genomic DNA.

With our remaining data, we sought to determine if the microbial *cpn*60 sequences we obtained were actually proportional and representative of the species present in the samples, independent of the non-specific amplification. To that end, we chose seven organisms that represented the most abundant species in the dataset (13-19% of the sequence data), species representing 1-2% of the data and species representing less than 1% of the data (Table 2). These species were specifically targeted and quantified by qPCR. Reassuringly, the sequence abundance data generated with *cpn*60 profiling correlated strongly with absolute species quantification by targeted qPCR (Table 2). Methodologically, this was very important and gives confidence in the analyses based on the read abundance values. Many metagenomic profiling studies draw conclusions from sequence abundance data but findings are never validated with another method.

## Conclusion

Investigation of microbiomes from A(H1N1) pdm09 patients showed that patient gender, originating healthy authority, sample type collected or sample designation as STAT/non-STAT were not consistently associated with upper respiratory tract microbiome structure. There was a trend for patients to have moderately more richness and diversity in their microbiomes with increasing age (particularly between patients younger and older than 20 years). At the phylum level, A(H1N1) pdm09 patients had microbiomes composed of Actinobacteria, Firmicutes and Proteobacteria while at the species level, microbiome compositions fell into one of 11 clusters dominated by one or two organisms. Finally, the *cpn*60 sequence read abundances generated in this study were validated by targeted qPCR of a select group of prevalent species.

## Supporting Information

Table S1 Upper respiratory tract sample descriptions.
(XLSX)

Table S2 OTU frequencies in all samples.
(XLSX)

Table S3 Frequencies of nearest neighbor "species" in all samples.
(XLSX)

Table S4 ANOVA analysis of diversity statistics.
(XLSX)

Figure S1 Principal coordinates analysis (PCoA) on Bray-Curtis and Canberra distance matrices.
(PDF)

Figure S2 Clustering of samples based on Unifrac distances.
(PDF)

Figure S3 Phylogenetic tree of all nearest neighbor "species" detected in upper respiratory tract microbiomes.
(PDF)
