# Supplementary material for: Characterization of the Upper Respiratory Tract Microbiomes of Patients with Pandemic H1N1 Influenza
Source: PLoS One. 2013 Jul 2;8(7):e69559. doi: 10.1371/journal.pone.0069559 (PMC3699515; doi:10.1371/journal.pone.0069559)
Supplement: Figure S1 — Principal coordinates analysis (PCoA) on Bray-Curtis and Canberra distance matrices. (PDF) [file pone.0069559.s005.pdf]

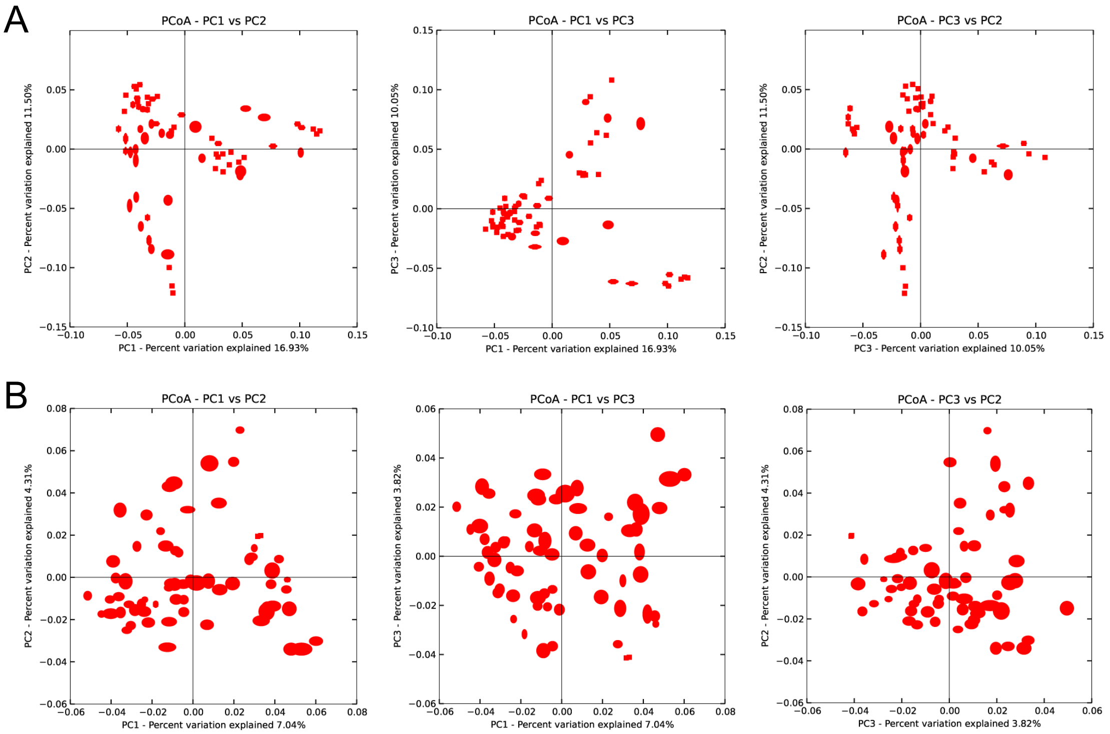

Figure S1 - Jackknifed principal coordinates analysis (PCoA) results on (A) Bray-Curtis distance matrices and (B) Canberra distance matrices. The area of each point indicates the interquartile range of the jackknifed PCoA estimates.
