# Supplementary material for: Characterization of the Upper Respiratory Tract Microbiomes of Patients with Pandemic H1N1 Influenza
Source: PLoS One. 2013 Jul 2;8(7):e69559. doi: 10.1371/journal.pone.0069559 (PMC3699515; doi:10.1371/journal.pone.0069559)
Supplement: Figure S2 — Clustering of samples based on Unifrac distances. (PDF) [file pone.0069559.s006.pdf]

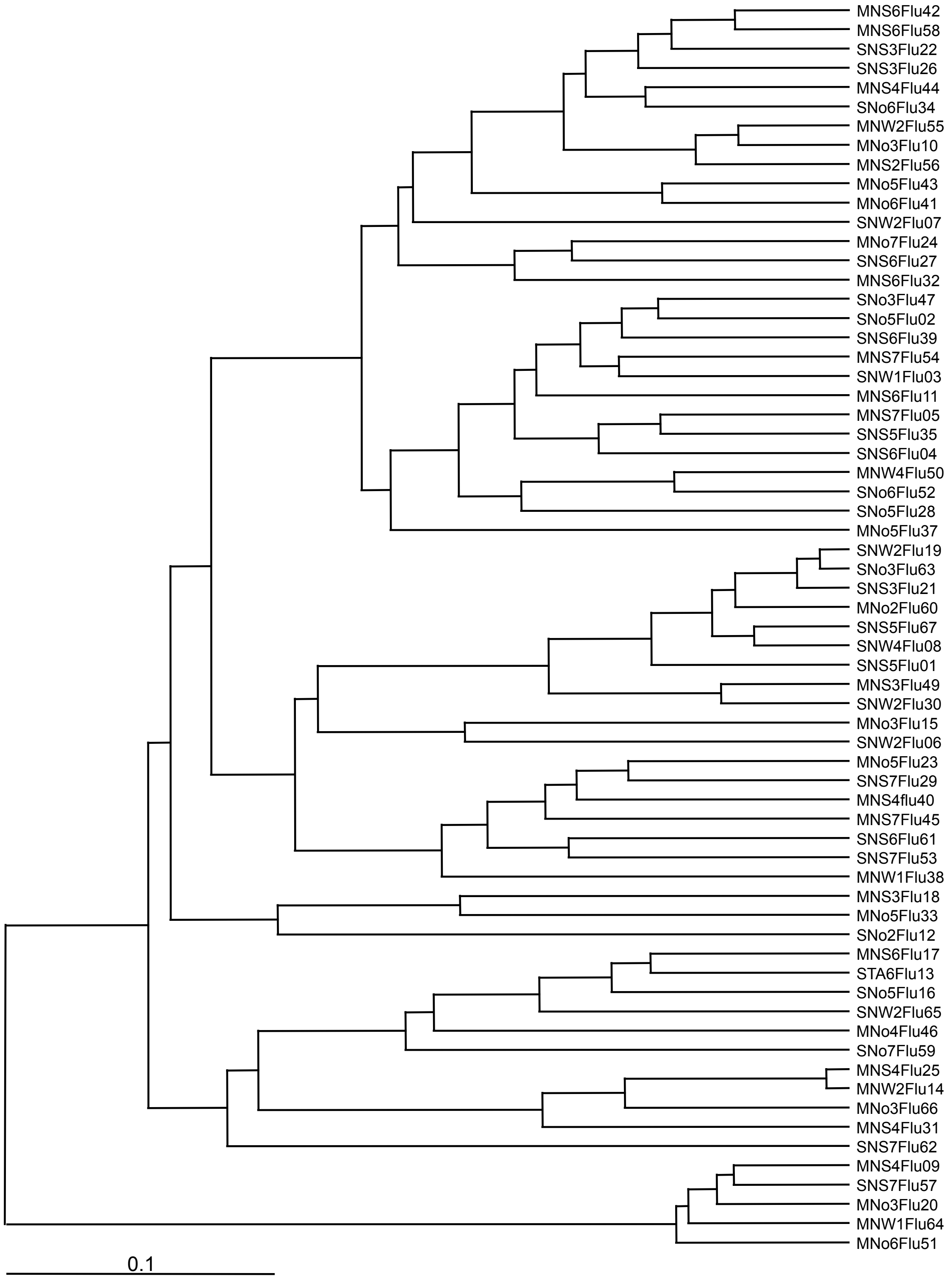


Figure S2. Clustering of nearest neighbour "species" by Unifrac distances. Sample label code: 1st letter represents sample designation (M = non-STAT; S = STAT); 2nd and 3rd letters represent specimen type (No = nasal swab, NS = nasopharyngeal swab, NW = nasopharyngeal washing, TA = tracheal aspirate); 4th number represents age group of patient (1 = 0.01-0.50 years, 2 = 0.51-3.0 years, 3 = 3.01 - 9.0 years, 4 = 9.01-20.0 years, 5 = 20.01-50.0 years, 6 = 50.01-65.0 years, 7 = >=65.01 years); Flu## represents the sample ID.
