# Supplementary material for: Characterization of the Upper Respiratory Tract Microbiomes of Patients with Pandemic H1N1 Influenza
Source: PLoS One. 2013 Jul 2;8(7):e69559. doi: 10.1371/journal.pone.0069559 (PMC3699515; doi:10.1371/journal.pone.0069559)
Supplement: Figure S3 — Phylogenetic tree of all nearest neighbor "species" detected in upper respiratory tract microbiomes. (PDF) [file pone.0069559.s007.pdf]

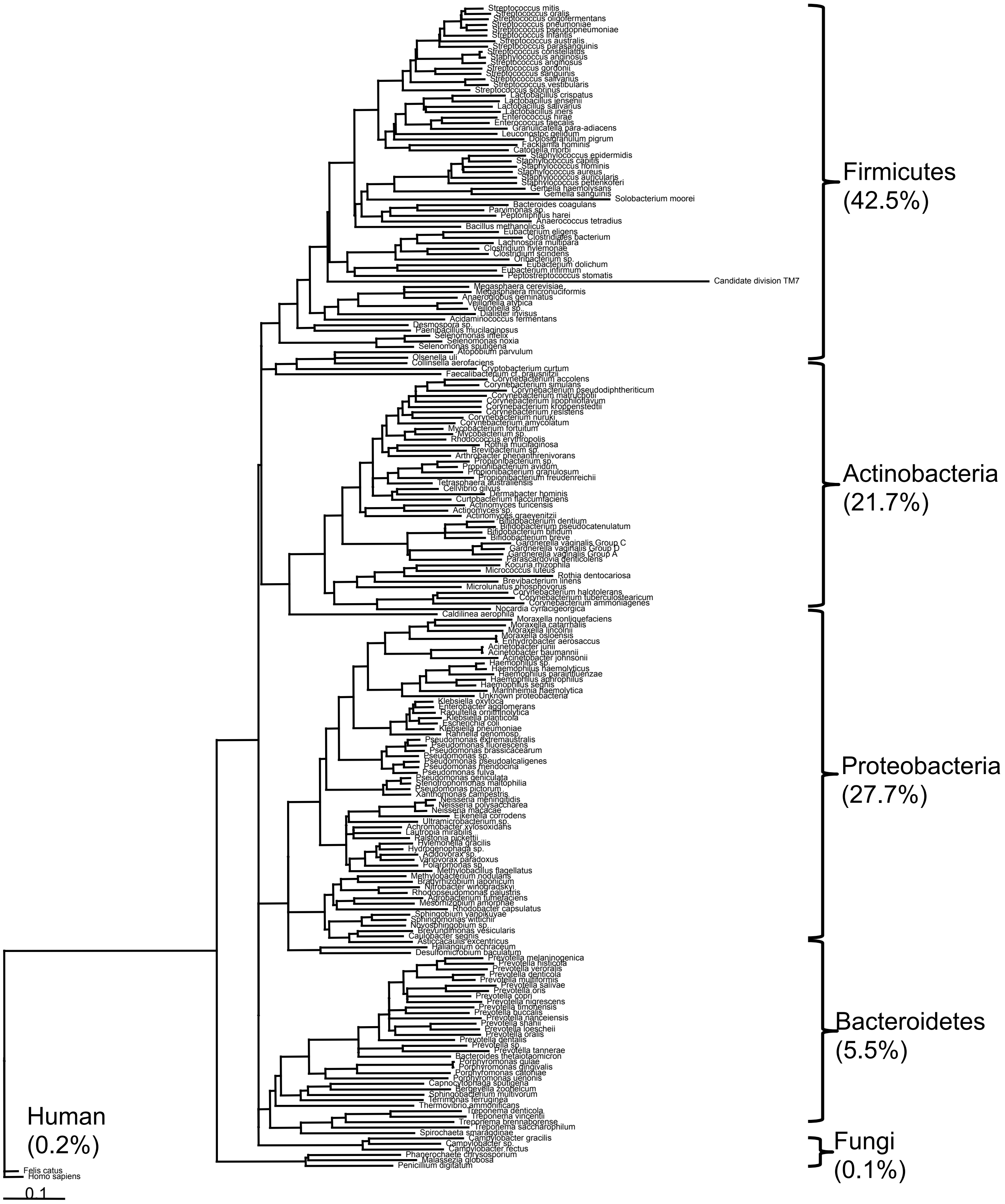

Figure S3 - Phylogenetic tree of all nearest neighbour "species" detected in upper respiratory microbiomes. Phyla are indicated with the proportion of the total dataset represented by each phylum indicated in parentheses.
